# Antiprotozoal activity of *Boesenbergia rotunda* (L.) Mansf and *Ganoderma lucidum* (Fr.) Kart extracts against *Blastocystis hominis*

**DOI:** 10.14202/vetworld.2023.187-193

**Published:** 2023-01-28

**Authors:** Chalermpon Kaewjai, Aunchalee Tonsomboon, Jaturawat Pawiwongchai, and Oranan Prommano

**Affiliations:** Faculty of Medical Technology, Rangsit University, Pathum Thani, Thailand

**Keywords:** antiprotozoal activity, *Blastocystis hominis*, *Boesenbergia rotunda*, *Ganoderma lucidum*

## Abstract

**Background and Aim:**

*Blastocystis* *hominis* is an intestinal protozoan in humans and animals. The parasite causes mild-to-severe intestinal complications, such as diarrhea, in healthy humans and immunocompromised hosts. This study aimed to determine the antiprotozoal activity of *Boesenbergia rotunda* (L.) Mansf and *Ganoderma lucidum* (Fr.) Kart extracts against *B. hominis*.

**Materials and Methods:**

Antiprotozoal activity of *B. rotunda* and *G. lucidum* extracts against *B. hominis* subtype 3 was determined using the erythrosin B exclusion assay, confirmed by a time-kill study. The morphology of the parasite treated with the extracts was observed by a scanning electron microscope. The phytochemicals present in *B. rotunda* and *G. lucidum* extracts were identified by gas chromatography-mass spectrometry analysis.

**Results:**

Both *B. rotunda* and *G. lucidum* extracts demonstrated strong antiprotozoal activity with similar minimum inhibitory concentration (MIC) values of 62.5 μg/mL. At 4× MIC and 8× MIC, both *B. rotunda* and *G. lucidum* extracts, and metronidazole inhibited the growth of *B. hominis* by up to 90% after 12 h treatment. *Blastocystis hominis* cells treated with *B. rotunda* extract, *G. lucidum* extract, and metronidazole were deformed and withered when compared with the control. Geraniol and versalide were found as the main compounds in *B. rotunda* and *G. lucidum* extracts, respectively.

**Conclusion:**

These results indicate the potential medicinal benefits of *B. rotunda* and *G. lucidum* extracts in the growth inhibition of *B. hominis*.

## Introduction

*Blastocystis hominis* is one of the most common intestinal protozoans detected in humans. The microorganism colonizes the intestines of humans and other animals, such as canids, swine, primates, rodents, and birds [1]. The role of *B. hominis* as a human pathogen is debatable. *Blastocystis* spp. can reportedly colonize the human intestine for a long time without causing any disease [2]. However, *Blastocystis* spp. can cause severe intestinal complications in humans through the fecal-oral route, zoonotically, and through waterborne transmission. The most frequent clinical signs of the disease are abdominal discomfort and diarrhea, which are followed by anorexia, fever, salivation, itching, and nausea [3]. Furthermore, the parasite causes diarrhea in immunocompromised hosts [4]. *Blastocystis hominis* is considered as a zoonotic microorganism. Numerous studies have been conducted globally on the frequency and genotyping of *Blastocystis* species in humans and animals and drinking water [5]. Importantly, non-human primates, artiodactyls, and birds may serve as reservoirs for human infection, especially in animal handlers [1]. The treatment of infection caused by *B. hominis* requires the consumption of drugs for a long duration, which may cause side-effects. In addition, resistance to metronidazole, a drug of choice, has been reported since 1976 [6]. Hence, the need to find an effective alternative drug to treat this parasite is great.

Medicinal plants based on their secondary metabolites have been used as an alternative treatment against protozoa. An ethanol extract of *Curcuma longa* rhizome possessed antimicrobial against *Acanthamoeba triangularis*, a keratitis-causing protozoan [7, 8]. *Kelussia odoratissima* Mozaff inhibited the growth of the promastigote and amastigote of *Leishmania major* [9]. Extracts from *Brucea javanica* seed and *Quercus infectoria* nut gall showed antiprotozoal activity against *B. hominis* [4]. This study focuses on the extracts of *Boesenbergia rotunda* (L.) Mansf rhizome and *Ganoderma lucidum* (Fr.) Kart fruiting body. *Boesenbergia* *rotunda*, previously known as *Boesenbergia pandurata*, common name Chinese ginger or Krachai-khao, belongs to the Zingiberaceae family and has been used in traditional medicine for the treatment of several infectious diseases, such as stomach discomfort and dysentery [10]. Interestingly, *B. rotunda* extract and its component panduratin A showed antiviral activity against severe acute respiratory syndrome coronavirus 2 (SARS-CoV-2) [11]. *Ganoderma lucidum*, common name Lingzhi or Reishi, belongs to the Ganodermataceae family and has been used in Chinese medicine for several purposes. The extract and pure compounds of *G. lucidum* exhibited antimicrobial activity against *Staphylococcus aureus*, *Pseudomonas aeruginosa*, *Plasmodium falciparum*, and *Candida albicans* [12].

To the best of our knowledge, no study on *B*. *rotunda* and *G. lucidum* extracts against *B. hominis* has been scientifically documented. Therefore, this study aimed to determine the antiprotozoal activity of *B. rotunda* and *G. lucidum* extracts against *B. hominis*. The antiprotozoal activity was determined using viability stain exclusion assays. The morphology of the parasite treated with the extracts was observed. Identification of the phytochemicals presented in *B. rotunda* and *G. lucidum* extracts was determined.

## Materials and Methods

### Ethical approval

All procedures in this study were approved by Exemption Ethical Committee of Research Institute of Rangsit University (Approval number RSPE 01/2560).

### Study period and location

The study was conducted from December 2020 to May 2021 and during November 2021. Preparation of the plant extraction, antiparasitic activity tests, and identification of the extracts were conducted at Rangsit University, Thailand.

### Cultivation of the parasite

A clinical isolate *B. hominis* subtype 3 was obtained from a medical laboratory department at the Faculty of Medical Technology, Rangsit University, Thailand. To perform *B. hominis* cultivation, Jones’ medium (2.65 g Na_2_HPO_4_·12H_2_O, 0.41 g KH_2_PO_4_, 7.36 g NaCl, 1.00 g yeast extract, and 950 mL deionized water) was used. A stock culture kept at −80°C was thawed and resuspended in Jones’ medium. The sample was centrifuged at 250× *g* for 5 min. The cell pellet was resuspended in Jones’ medium supplemented with 10% bovine serum without antibiotics. The parasite was incubated at 37°C under anaerobic conditions and subcultured every 48 h.

### Preparation of the plant extracts and an antimicrobial agent

Commercial *B. rotunda* rhizome ethanol extract and *G. lucidum* fruiting body aqueous extract were purchased from Asian Bioplex Company, Thailand. Briefly, 100 mg of each extract was dissolved in 10 mL of 100% dimethyl sulfoxide (DMSO) (Sigma, USA). The final concentration of the stock was 10 mg/mL. The samples were filtered through a sterile 0.45 mm filter (Sartorious, Germany) and kept at 4°C until use. Metronidazole (Sigma), an antibiotic of choice, was included as a positive control. The antibiotic was dissolved in 100% DMSO and kept at −20°C until use.

### Antiprotozoal activity of *B. rotunda* and *G. lucidum* extracts against *B. hominis*

**Figure-1:**
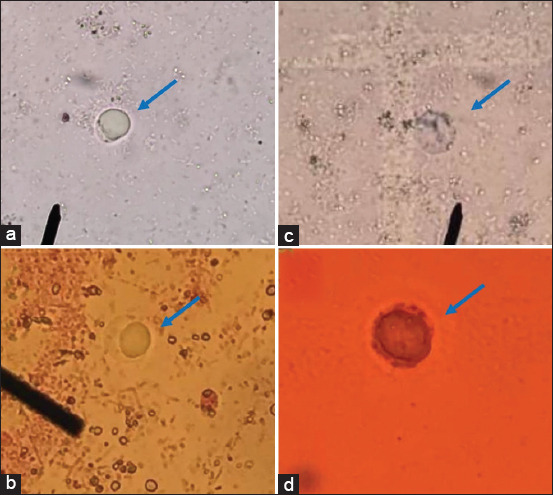
(a and b) Viable cells and (c and d) dead cells of *Blastocystis hominis* cells detected by (a and c) Trypan blule and (b and d) erythrosin B. The viable cells demonstrated non-stained cells, while the dead cells presented stained cells.

The preliminary screening of the antiprotozoal activity of 1000 μg/mL *B. rotunda* and *G. lucidum* extracts against *B. hominis* was evaluated by the erythrosin B exclusion assay, as described [13–15], with minor modification. Briefly, *B. hominis* cells were cultured for 48 h, harvested, and resuspended in Jones’ medium. An aliquot of 100 μL of the cell suspension (2 × 10^5^ trophozoites/mL) was dropped into 96-well plates, containing 100 μL of each extract at a concentration 1000 μg/mL. One percent DMSO was used as a negative control, whereas 20 μg/mL metronidazole was used as a positive control. The sample was incubated at 25°C for 24 h. The detection of dead *B. hominis* cells in the presence of antimicrobial agents was also confirmed by the Trypan blue exclusion assay. The inhibition of protozoan growth was determined using vital dyes to investigate the number of live (non-stained) and dead (stained) cells (Figure-1). The relative percentage of cell inhibition was defined as:

Percent inhibition = 100 − [(mean of the treated *B. hominis*/mean of the control) × 100]

Selection of the extracts was chosen when they showed ≥90% of growth inhibition.

The antiprotozoal activity of the plant extracts was further performed by determining the minimum inhibitory concentration (MIC) using the broth microdilution assay. Briefly, 100 μL of *B. hominis* suspension (2 × 10^5^ cells/mL) was added into 96-well plates containing 100 μL of successively diluted extract at concentrations 31.25–2000 μg/mL. Moreover, 1% of DMSO and metronidazole were used as negative and positive controls, respectively. The sample was incubated at 25°C for 24 h. The MIC value was specified as the lowest concentration that caused ≥90% growth inhibition (mean ± standard deviation [SD]), as described above.

### Time-kill study

The time-kill study of *B. rotunda* and *G. lucidum* extracts against *B. hominis* was investigated as described [16] with some modifications. Briefly, an inoculum (2 × 10^5^ colony-forming unit/mL) of the culture was added in Jones’ medium supplemented with each extract at concentrations of 1×, 2×, 4×, and 8× MIC. One percent DMSO and metronidazole were used as negative and positive controls, respectively. The samples were incubated at 37°C and sampled to viable counts performed at different times (12, 24, 48, and 72 h) using the erythrosin B exclusion assay. The relative percentage of cell inhibition was calculated as described above.

### Morphology of *B. hominis* after treatment with the plant extracts

The morphology of *B. hominis* after the treatment with the plant extracts was observed by a scanning electron microscope (SEM) as described [17] with some modifications. *Blastocystis hominis* cells were treated with the extracts at 8× MIC on a sterile glass coverslip in a 24-well plate for 24 h. Subsequently, the solution was removed and the samples were rinsed thrice with phosphate buffer solution (PBS, pH 7.2). The samples were then fixed with glutaraldehyde at a concentration of 2.5% in PBS for 24 h, and washed twice with PBS. The samples were dehydrated in a series of graded ethanol (20%–100%). The samples were then dehydrated using a critical point dryer and coated with gold particles. The morphology (size, shape, and structure) of *B. hominis* post-treatment was observed using the JEOL JSM-5410LV (SEM, Japan).

### Phytochemicals in the extracts of *B. rotunda* and *G. lucidum*

Phytochemical constituents in the extracts of *B. rotunda* and *G. lucidum* were analyzed using gas chromatography-mass spectrometry (GC-MS) (Agilent GC 7890A gas chromatography system equipped with 5975C inert XL EI/CI MSD with triple-Axis Detector). Briefly, a DB-5MS column of dimensions 30 m ×250 μm × 0.25 μm was used with helium gas as a carrier at a flow rate of 1 mL/min. The column temperature was initially programmed at 60°C, and increased to 160°C at 10°C/min. Then, the temperature was further increased to 250°C at 2.5°C/min and held for 15 min. Mass spectrometry was performed in the electron ionization mode at 70 eV, with a source temperature of 230°C, with continuous scanning from 35 to 500 m/z. The chemical constituents in the herbal extracts, including the oil and crude extract, were identified by comparing their mass spectral data with those from the Wiley library.

### Statistical analysis

All experiments were carried out in triplicate. The results were presented as mean ± SD. All data were recorded, and entered using R program version 4.2.2 (www.r-project.org, Free software foundation Inc., USA). The median of each group was compared using the Kruskal–Wallis test. Then, Dunn’s test was used to determine a significant pair. Differences were considered significant at p < 0.05.

## Results

### Antiprotozoal activity of *B. rotunda* and *G. lucidum* extracts against B. hominis

The inhibition of *B. hominis* cell growth by antimicrobial agents was detected using Trypan blue and erythrosin B. Viable cells were unstained (Figures-1a and b), whereas dead cells were stained (Figures-1c and 1d). Both *B. rotunda* and *G. lucidum* extracts at a concentration of 1000 μg/mL showed antiprotozoal activity against *B. hominis* (Table-1). Both *B. rotunda* and *G. lucidum* extracts demonstrated strong antiprotozoal activity with similar MIC values of 62.5 μg/mL. The MIC value of metronidazole against the parasite was 1.25 μg/mL.

**Table-1:** Screening of antiparasitic activity and minimal inhibitory concentration of *B. rotunda* and *G. lucidum* extracts against *B. hominis.*

Agents	Screening of antiparasitic activity at 24 h (1000 µg/mL)	MIC (µg/mL)
*B. rotunda* extract	+	62.5
*G. lucidum* extract	+	62.5
Metronidazole^a^	+	1.25

a The concentration of metronidazole for the screening was 20 μg/mL. *B. rotunda*=*Boesenbergia rotunda*, *G. lucidum*=*Ganoderma lucidum*, *B. hominis=Blastocystis hominis.*

### Time-kill study

**Figure-2:**
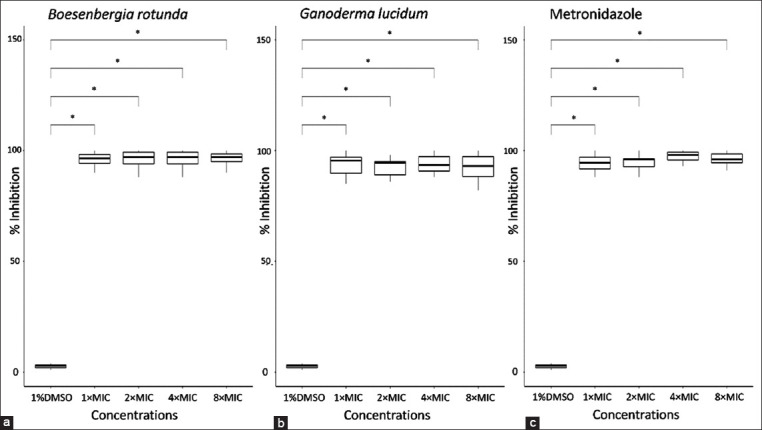
Box plot of the growth inhibition of *Blastocystis hominis* after the treatment with (a) *Boesenbergia rotunda* extract, (b) *Ganoderma lucidum* extract, and (c) metronidazole, compared with the control (1% dimethyl sulfoxide). A significant difference was considered at p < 0.05 (*).

To confirm the effectiveness of *B. rotunda* and *G. lucidum* extracts against *B. hominis*, a time-kill study of the extracts at different concentrations based on MIC was performed. The antimicrobial activity of *B. rotunda* and *G. lucidum* extracts was concentration dependent, resulting in the growth inhibition of *B. hominis* cells (Table-2). Approximately 90% of growth inhibition was observed when the parasite was treated with *B. rotunda* at 1× MIC for 12 h, same as the positive control metronidazole. At the same concentration, approximately 93% of growth inhibition was detected after the challenge with *G. lucidum* at 1× MIC for 24 h. At 4× and 8× MIC, the *B. rotunda* extract, *G. lucidum* extract, and metronidazole inhibited the growth of *B. hominis* by up to 90% after 12 h treatment. In addition, the percent inhibition of all concentrations tested for both extracts and metronidazole was significantly different compared with the control (Figures-2a–c).

**Table-2:** Percent inhibition *B. hominis* growth after treatment by *B. rotunda* and *G. lucidum* extracts at different time points.

Agents/concentrations	Times (h)			

	12	24	48	72
*B. rotunda* extract				
8 × MIC	92.50 ± 3.54	97.22 ± 3.93	98.00 ± 2.83	97.37 ± 3.72
4 × MIC	91.67 ± 5.89	97.06 ± 4.16	98.86 ± 1.61	96.88 ± 4.42
2 × MIC	90.63 ± 4.42	97.50 ± 3.54	98.21 ± 2.53	98.53 ± 2.08
1 × MIC	90.38 ± 2.72	97.92 ± 2.95	95.95 ± 1.91	98.57 ± 2.02
*G. lucidum* extract				
8 × MIC	90.91 ± 3.21	96.51 ± 1.64	93.75 ± 2.95	97.92 ± 2.95
4 × MIC	92.22 ± 1.57	93.55 ± 4.56	93.48 ± 3.07	97.50 ± 3.54
2 × MIC	85.71 ± 0.00	93.90 ± 5.17	94.87 ± 3.63	95.24 ± 0.00
1 × MIC	87.50 ± 3.54	93.42 ± 5.58	95.95 ± 1.91	98.28 ± 2.44
Metronidazole				
8 × MIC	90.38 ± 8.16	93.18 ± 3.21	98.00 ± 2.83	97.73 ± 3.21
4 × MIC	90.38 ± 2.72	96.88 ± 2.21	99.06 ± 1.33	97.50 ± 3.54
2 × MIC	94.23 ± 2.72	96.00 ± 0.00	91.67 ± 5.89	98.44 ± 2.21
1 × MIC	94.12 ± 4.16	95.16 ± 2.28	91.67 ± 5.89	98.44 ± 2.21

*B. rotunda*=*Boesenbergia rotunda*, *G. lucidum*=*Ganoderma lucidum*, *B. hominis=Blastocystis hominis*, MIC=Minimum inhibitory concentration

### Morphology of *B. hominis* after the treatment with the extracts

**Figure-3:**
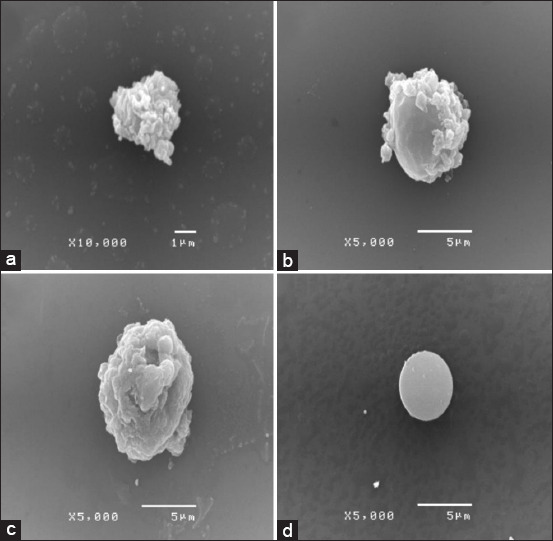
Morphology of *Blastocystis hominis* cells after treatment with (a) *Boesenbergia rotunda* extract, (b) *Ganoderma lucidum* extract, and (c) metronidazole as observed by scanning electron microscope. (d) About 1% of dimethyl sulfoxide was used as the negative control.

The morphology of *B. hominis* cells after the treatment with *B. rotunda* extract and *G. lucidum* extract was observed by SEM. The control cells had an oval shape and a smooth surface (Figure-3d). *Blastocystis hominis* cells treated with *B. rotunda* extract (Figure-3a), *G. lucidum* extract (Figure-3b), and metronidazole (Figure-3c) at 8× MIC were deformed, with withered shapes, when compared with the control. This shrinking of cells was dramatic when the cells were challenged with *B. rotunda* extract, *G. lucidum* extract, and metronidazole.

### Phytochemicals in the extracts of *B. rotunda* and *G. lucidum*

The phytochemicals in the extracts of *B. rotunda* and *G. lucidum* were investigated by GC-MS analysis. Geraniol was the main compound in *B. rotunda* extract, followed by camphor, methyl cinnamate, and linalool. In *G. lucidum* extract, versalide was the main compound, followed by astratone. The percentage and structure of each compound are presented in Table-3.

**Table-3:** Phytochemicals in the extracts of *B. rotunda* and *G. lucidum.*

Plant extract/compounds	Types of compounds	Percentage of compounds (%)	Structures
*B. rotunda*			
Geraniol	Terpenoids	27.63	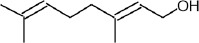
Camphor	Terpenoids	12.92	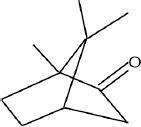
Methyl cinnamate	Benzene derivatives	6.98	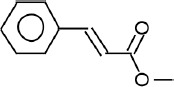
Linalool	Terpenoids	0.69	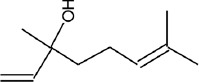
*G. lucidum*			
Versalide	Benzene derivatives	54.39	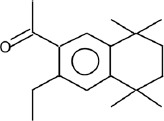
Astratone	Macrolides	45.61	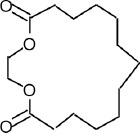

*B. rotunda*=*Boesenbergia rotunda*, *G. lucidum*=*Ganoderma lucidum*

## Discussion

*Blastocystis hominis* infection in humans is commonly linked to poor sanitation, living in tropical or subtropical climates, exposure to diseased animals, and consuming contaminated food or water [18]. The distribution of the parasite is worldwide, especially in tropical and subtropical areas, including South-east Asia. *Blastocystis* spp. can cause severe intestinal complications, such as diarrhea, in healthy humans and immunocompromised hosts. This study is focused on the antiprotozoal activity of *B. rotunda* extract and *G. lucidum* extract against the clinical isolate *B. hominis* subtype 3. The results showed that both *B. rotunda* and *G. lucidum* extracts demonstrated antiparasitic activity against the pathogen. Studying stool samples under a light microscope is the most common method of diagnosing the parasite. Stained samples using vital dyes are recommended to differentiate between viable and dead cells [3]. Trypan blue exclusion test is used to determine the number of viable cells. It is based on the principle that live cells possess intact cell membranes that exclude certain dyes, whereas dead cells do not [14]. Alternately, erythrosin B stain, a non-toxic viability dye, is used to assess cell viability. Viable cells present intact cell membranes and are not stained. Furthermore, erythrosin B has been proven to be preferable than Trypan blue in measuring cell viability [15]. The dead cells were dyed cherry pink by erythrosin B and were easy to detect.

The findings of this study demonstrated the antiprotozoal activity of *B. rotunda* and *G. lucidum* extracts against *B. hominis*. The plant species have been used as a traditional medicine in tropical countries for the treatment of diarrhea and intestinal disorders. Chloroform and methanol extracts of *B. rotunda* rhizome exhibit antiparasitic activity against *Entamoeba histolytica* [19] and *Giardia intestinalis* [20]. In addition, *B. rotunda* extracts demonstrated antimicrobial activity against *S. aureus*, *S. epidermidis*, *Bacillus subtilis*, and *C. albicans*. The topical application of the *B. rotunda* extract significantly increased wound healing [21]. Bioactive compounds, such as essential oils, terpenoids, and flavonoids, were found in the rhizome of *B. rotunda* [22], which is consistent with the findings of this study. Recently, *B. rotunda* extract and its component panduratin A were shown to demonstrate antiviral activity against SARS-CoV-2 [11]. Furthermore, *B. rotunda* extract inhibited tumor necrosis factor-α-induced cytotoxicity in L929 cells [23]. The anticancer activity of gingerol has been the most active gradient effect on cancer cells. Also, geraniol was given lower glucose and urea in rats [24, 25]. This study revealed that geraniol, a terpenoid compound, was the main compound detected in the ethanol extract of *B. rotunda* rhizome, followed by camphor and methyl cinnamate. To the best of our knowledge, no study has documented the effect of pure geraniol, camphor, and methyl cinnamate against the parasite.

*Ganoderma lucidum*, a well-known medicinal plant, has been consumed for several years in Asian countries due to its many health benefits. The extract of *G. lucidum* and its pure compounds showed antimicrobial activity against bacteria, parasites, and yeasts [12]. The antimalarial activity of *G. lucidum* has been proposed by many studies [26, 27]. Terpenoid extracts from *G. lucidum* fruit bodies demonstrated antimalarial activity by a mechanism involving a reduction in erythrocyte and hepatic lipids in *Plasmodium berghei* [26]. Furthermore, terpenoid extracts of *Ganoderma* protected the liver against *P. berghei*-induced damage in infected mice [26]. The findings of this study showed that versalide is the main compound in *G. lucidum* extract followed by astratone. However, no study on pure compounds of versalide and astratone against the parasite has been scientifically reported. This study used metronidazole as the positive control in all experiments. Metronidazole is active against a variety of protozoa and bacteria. The drug diffuses into the organism, inhibits protein synthesis by interacting with DNA, and causes a loss of helical DNA structure and strand breakage. Therefore, it causes cell death in susceptible organisms [28]. *Blastocystis hominis* cells treated with *B. rotunda* extract, *G. lucidum* extract, and metronidazole showed deformity in the form of withered shapes, when compared with the control. Furthermore, the shrinking of cells was dramatic when the cells were challenged with *G. lucidum* extract and metronidazole.

The limitations of this study include the isolation of pure compounds from *B. rotunda* and *G. lucidum* extract. The antiparasitic mechanisms of action of both extracts and pure compounds against *B. hominis* should be determined. In the future, *in vivo* or *ex vivo* studies should investigate the mechanism of action of *B. rotunda* and *G. lucidum* extracts against *B. hominis* in animals. Importantly, the cytotoxicity of both the plant extracts should be determined against human and animal cell lines.

## Conclusion

This study presented that *B. rotunda* and *G. lucidum* extracts inhibited the growth of *B. hominis* subtype 3 with similar MIC values of 62.5 μg/mL. At 4× and 8× MIC, both *B. rotunda* extract and *G. lucidum* extract and metronidazole inhibited the growth of *B. hominis* by up to 90% after 12 h treatment. *Blastocystis hominis* cells treated with *B. rotunda* extract, *G. lucidum* extract, and metronidazole showed deformity in the form of withered shapes, when compared with the control. Geraniol was found as the main compound in *B. rotunda* extract, whereas versalide was the main compound in *G. lucidum* extract. These findings suggest the potential medicinal benefits of *B. rotunda* and *G. lucidum* extracts in the growth inhibition of *B. hominis*.

## Authors’ Contributions

CK: Designed the experiments, performed the experiments, analyzed and interpreted the data, and wrote the manuscript. AT and JP: Designed the experiment protocol. OP: Given the valuable concept during the experiment. All authors have read and approved the final manuscript.

## References

[1] Cian A, El Safadi D, Osman M, Moriniere R, Gantois N, Benamrouz-Vanneste S, Delgado-Viscogliosi P, Guyot K, Li L.L, Monchy S (2017). Molecular epidemiology of *Blastocystis* spp. in various animal groups from two French zoos and evaluation of potential zoonotic risk. PLoS One.

[2] Scanlan P.D, Stensvold C.R, Rajilić-Stojanović M, Heilig H.G, De Vos W.M, O'Toole P.W, Cotter P.D (2014). The microbial eukaryote *Blastocystis* is a prevalent and diverse member of the healthy human gut microbiota. FEMS Microbiol. Ecol.

[3] Beyhan Y.E, Yilmaz H, Cengiz Z.T, Ekici A (2015). Clinical significance and prevalence of *Blastocystis hominis* in Van, Turkey. Saudi Med. J.

[4] Sawangjaroen N, Sawangjaroen K (2005). The effects of extracts from anti-diarrheic Thai medicinal plants on the *in vitro* growth of the intestinal protozoa parasite:*Blastocystis hominis*. J. Ethnopharmacol.

[5] Lee L.I, Chye T.T, Karmacharya B.M, Govind S.K (2012). *Blastocystis* spp.:Waterborne zoonotic organism, a possibility?. Parasit. Vectors.

[6] Younis M.S, Abououf E.A, El Saeed Ali A, Wassef R.M (2020). *In vitro* effect of silver nanoparticles on *Blastocystis hominis*. Int. J. Nanomedicine.

[7] Mitsuwan W, Bunsuwansakul C, Leonard T.E, Laohaprapanon S, Hounkong K, Bunluepuech K, Kaewjai C, Mahboob T, Samudi C, Dhobi M, Pereira M.L, Nawaz M, Wiart C, Nissapatorn V (2020). *Curcuma longa* ethanol extract and Curcumin inhibit the growth of *Acanthamoeba triangularis* trophozoites and cysts isolated from water reservoirs at Walailak University, Thailand. Pathog. Glob. Health.

[8] Mitsuwan W, Sangkanu S, Romyasamit C, Kaewjai C, Jimoh T.O, Pereira M.L, Siyadatpanah A, Kayesth S, Nawaz M, Rahamatullah M, Butler M.S, Wilairatana P, Wiart C, Nissapatorn V (2020). *Curcuma longa* rhizome extract and Curcumin reduce the adhesion of *Acanthamoeba triangularis* trophozoites and cysts in polystyrene plastic surface and contact lens. Int. J. Parasitol. Drugs Drug Resist.

[9] Mirzaei F, Siyadatpanah A, Mitsuwan W, Nissapatorn V, Nilforoushzadeh M, Maleksabet A, Hosseini M, Pereira M.L, Hejazi S.H (2020). Butanol fraction of *Kelussia odoratissima* Mozaff inhibits the growth of *Leishmania major* promastigote and amastigote. Worlds Vet. J.

[10] Salama S.M, Bilgen M, Al Rashdi A.S, Abdulla M.A (2012). Efficacy of *Boesenbergia rotunda* treatment against thioacetamide-induced liver cirrhosis in a rat model. Evid. Based Complement. Alternat. Med.

[11] Kanjanasirirat P, Suksatu A, Manopwisedjaroen S, Munyoo B, Tuchinda P, Jearawuttanakul K, Seemakhan S, Charoensutthivarakul S, Wongtrakoongate P, Rangkasenee N, Pitiporn S, Waranuch N, Chabang N, Khemawoot P, Sa-Ngiamsuntorn K, Pewkliang Y, Thongsri P, Chutipongtanate S, Hongeng S, Borwornpinyo S, Thitithanyanont A (2020). High-content screening of Thai medicinal plants reveals *Boesenbergia rotunda* extract and its component Panduratin A as anti-SARS-CoV-2 agents. Sci. Rep.

[12] Basnet B.B, Liu L, Bao L, Liu H (2017). Current and future perspective on antimicrobial and anti-parasitic activities of *Ganoderma* spp.:An update. Mycology.

[13] Mitsuwan W, Sin C, Keo S, Sangkanu S, Pereira M.L, Jimoh T.O, Salibay C.C, Nawaz M, Norouzi R, Siyadatpanah A, Wiart C, Wilairatana P, Mutombo P.N, Nissapatorn V (2021). Potential anti-*Acanthamoeba* and anti-adhesion activities of *Annona muricata* and *Combretum trifoliatum* extracts and their synergistic effects in combination with chlorhexidine against *Acanthamoeba triangularis* trophozoites and cysts. Heliyon.

[14] Strober W (2015). Trypan blue exclusion test of cell viability. Curr. Protoc. Immunol.

[15] Krause A.W, Carley W.W, Webb W.W (1984). Fluorescent erythrosin B is preferable to trypan blue as a vital exclusion dye for mammalian cells in monolayer culture. J. Histochem. Cytochem.

[16] Mitsuwan W, Jiménez-Munguía I, Visutthi M, Sianglum W, Rodríguez-Ortega M.J, Voravuthikunchai S.P, REIPI/GEIH Study Group (2019). Rhodomyrtone decreases *Staphylococcus aureus* SigB activity during exponentially growing phase and inhibits haemolytic activity within membrane vesicles. Microb. Pathog.

[17] Kulnanan P, Chuprom J, Thomrongsuwannakij T, Romyasamit C, Sangkanu S, Manin N, Nissapatorn V, de Lourdes Pereira M, Wilairatana P, Kitpipit W, Mitsuwan W (2022). Antibacterial, antibiofilm, and anti-adhesion activities of *Piper betle* leaf extract against Avian pathogenic *Escherichia coli*. Arch. Microbiol.

[18] Duda A, Kosik-Bogacka D, Lanocha-Arendarczyk N, Kołodziejczyk L, Lanocha A (2015). The prevalence of *Blastocystis hominis* and other protozoan parasites in soldiers returning from peacekeeping missions. Am. J. Trop. Med. Hyg.

[19] Sawangjaroen N, Phongpaichit S, Subhadhirasakul S, Visutthi M, Srisuwan N, Thammapalerd N (2006). The anti-amoebic activity of some medicinal plants used by AIDS patients in southern Thailand. Parasitol. Res.

[20] Sawangjaroen N, Subhadhirasakul S, Phongpaichit S, Siripanth C, Jamjaroen K, Sawangjaroen K (2005). The *in vitro* anti-giardial activity of extracts from plants that are used for self-medication by AIDS patients in southern Thailand. Parasitol. Res.

[21] Jitvaropas R, Saenthaweesuk S, Somparn N, Thuppia A, Sireeratawong S, Phoolcharoen W (2012). Antioxidant, antimicrobial and wound healing activities of *Boesenbergia rotunda*. Nat. Prod. Commun.

[22] Rosdianto A.M, Puspitasari I.M, Lesmana R, Levita J (2020). Bioactive compounds of *Boesenbergia* spp. and their anti-inflammatory mechanism:A review. J. Appl. Pharm. Sci.

[23] Morikawa T, Funakoshi K, Ninomiya K, Yasuda D, Miyagawa K, Matsuda H, Yoshikawa M (2008). Medicinal foodstuffs. XXXIV. Structures of new prenylchalcones and prenylflavanones with TNF-a and aminopeptidase N inhibitory activities from *Boesenbergia rotunda*. Chem. Pharm. Bull.

[24] Farhath M.S, Vimal M (2012). An evaluation of toxicity in essential oils of geraniol, geranial acetate, gingerol and eugenol in rats. Int. J. Phytomed.

[25] Plengsuriyakarn T, Viyanant V, Eursitthichai V, Tesana S, Chaijaroenkul W, Itharat A, Na-Bangchang K (2012). Cytotoxicity, toxicity, and anticancer activity of *Zingiber officinale* Roscoe against cholangiocarcinoma. Asian Pac. J. Cancer Prev.

[26] Oluba O.M (2019). *Ganoderma* terpenoid extract exhibited anti-plasmodial activity by a mechanism involving reduction in erythrocyte and hepatic lipids in *Plasmodium berghei* infected mice. Lipids Health Dis.

[27] Adams M, Christen M, Plitzko I, Zimmermann S, Brun R, Kaiser M, Hamburger M (2010). Antiplasmodial lanostanes from the *Ganoderma lucidum* mushroom. J. Nat. Prod.

[28] Löfmark S, Edlund C, Nord C.E (2010). Metronidazole is still the drug of choice for treatment of anaerobic infections. Clin. Infect. Dis.

